# Health-related quality of life in patients with pulmonary arterial hypertension

**DOI:** 10.1186/1465-9921-6-92

**Published:** 2005-08-10

**Authors:** Darren B Taichman, Jennifer Shin, Laryssa Hud, Christine Archer-Chicko, Sandra Kaplan, Jeffery S Sager, Robert Gallop, Jason Christie, John Hansen-Flaschen, Harold Palevsky

**Affiliations:** 1 Pulmonary, Allergy and Critical Care Division, University of Pennsylvania School of Medicine, University of Pennsylvania Medical Center-Presbyterian, Philadelphia, PA 19104, USA

## Abstract

**Background:**

Improved outcomes with expanding treatment options for patients with pulmonary arterial hypertension present the opportunity to consider additional end-points in approaching therapy, including factors that influence health-related quality of life. However, comparatively little is known about health-related quality of life and its determinants in patients with pulmonary arterial hypertension.

**Methods:**

Health-related quality of life was evaluated in a cross sectional study of 155 outpatients with pulmonary arterial hypertension using generic and respiratory-disease specific measurement tools. Most patients had either World Health Organization functional Class II or III symptoms. Demographic, hemodynamic and treatment variables were assessed for association with health-related quality of life scores.

**Results:**

Patients with pulmonary arterial hypertension suffered severe impairments in both physical and emotional domains of health-related quality of life. Patients with idiopathic ("primary") pulmonary arterial hypertension had the best, and those with systemic sclerosis the worst health-related quality of life. Greater six-minute walk distance correlated with better health-related quality of life scores, as did functional Class II versus Class III symptoms. Hemodynamic measurements, however, did not correlate with health-related quality of life scores. No differences in health-related quality of life were found between patients who were being treated with calcium channel antagonists, bosentan or continuously infused epoprostenol at the time of quality of life assessment.

**Conclusion:**

Health-related quality of life is severely impaired in patients with pulmonary arterial hypertension and is associated with measures of functional status. Specific associations with impaired health-related quality of life suggest potential areas for targeted intervention.

## Introduction

Pulmonary arterial hypertension (PAH) is a devastating disease, characterized by progressive dyspnea and exercise limitation. If not effectively controlled, PAH often progresses to right heart failure and premature death [1-3]. Fortunately, recent dramatic advances in pharmacological treatment have brought about significant improvements in physical functioning and survival, and new therapeutic options are emerging at a rapid pace [4]. Improved outcomes with expanding treatment options present the opportunity to consider additional end-points in choosing approaches to therapy, including factors influencing health-related quality of life.

Presently, little is known about the determinants of health-related quality of life (HRQOL) in patients with PAH. An improved understanding of these determinants will be essential as HRQOL becomes an important outcome in research on therapeutics for PAH [5,6].We studied health-related quality of life in a large population of PAH patients and performed an initial assessment of the clinical factors associated with better (or worse) HRQOL.

## Methods

### Study Design and Patients

We conducted a cross sectional survey of HRQOL in patients cared for in the Pulmonary Vascular Disease Program at the University of Pennsylvania Health System. Previously established as well as newly referred patients with PAH were asked to complete HRQOL questionnaires over one year (October, 2002 – September, 2003). PAH was defined according to standard criteria, including a mean pulmonary artery pressure >25 mmHg at rest or 30 mmHg with exertion, and the absence of significant left heart dysfunction [7,8]. Patients completed questionnaires without assistance at regularly scheduled appointments prior to the physician evaluation. Physicians and nurses were not aware of HRQOL survey responses.

Patient demographics, symptoms, medications, and physical exam findings on the day of HRQOL evaluations were obtained by chart review, as were demographic values. Test results within three months of HRQOL assessment were recorded (e.g. hemodynamic values from cardiac catheterization in 103 patients and six-minute walk distance in 33 patients).

### Health-Related Quality of Life Measurements

We administered a generic and a respiratory disease-specific quality of life questionnaire. The Medical Outcome Study 36 Item Short Form health survey (SF-36, standard form, version 2; Quality Metric, Lincoln, RI) yields scaled scores in 8 physical and mental health areas affected by health and disease conditions, in addition to physical component and mental component summary scores [9]. The St. George's Respiratory Questionnaire (SGRQ) is a well validated respiratory disease specific HRQOL measure, yielding scores related to symptoms (concerning the frequency and severity of respiratory symptoms), activity (the degree to which activities are limited by breathlessness), and impact (aspects of social and psychological function affected by respiratory disease). In addition, a total score summarizes disease impact on overall health status [10].

### Statistical Analysis

Scores for the 8 domains and 2 summary measures of the SF-36, and the 4 scores of the SGRQ were calculated electronically according to published guidelines, including imputation for missing responses [9]. Higher SF-36 scores indicate better quality of life, whereas higher SGRQ scores indicate poorer HRQOL. SF-36 scores are normalized to a mean of 50 and standard deviation of 10, based on the normal US population. Mean raw scores on the SGRQ were compared with established normal population scores [11].

Data were entered into a Microsoft Access 5.1 database (Microsoft Corporation) and statistical analysis performed with SAS 8.2 software (SAS Institute, Cary, NC). Data were analyzed for differences using an independent t-test for binary predictors, ANOVA for categorical predictors, and correlation coefficients for continuous measures. Comparisons between patient groups were limited to the physical and mental component summary scores of the SF-36, and significant associations defined as those having a p ≤ 0.05.

Normalized physical and mental component summary scores were calculated from published studies of populations with various chronic diseases using reported individual SF-36 domain scores and an on-line scoring program .

This study was approved by the Institutional Review Board of the University of Pennsylvania.

## Results

### Patient Characteristics

Health-related quality of life was examined in 155 adult outpatients. The population was 81% female with an average age of 53 years, ranging from 18 to 84. Other physical and social characteristics of the study population are shown in Table 1. Thirty-three patients (21% of the sample) completed HRQOL questionnaires at the time of initial evaluation in a pulmonary vascular disease specialty program. Patients who had been followed for treatment of PAH for up to 1, 2 or 3 years accounted for 19, 25 and 14 percent of evaluations, respectively; the remaining 21% of patients had been followed longer than 3 years. Approximately one third of patients had idiopathic ("primary") pulmonary arterial hypertension and most had either World Health Organization Class II or III symptoms. The majority were married and only 14 percent lived alone. Thirty-five percent of the patients were employed.

**Table 1:** Baseline Characteristics

**Physical Characteristics**		**Social Characteristics**	
Patients, n	155	Marital Status	
Age, years (mean ± SD)	53 ± 13	Single	25 (16)
Female, n (%)	126 (81)	Married	100 (65)
Caucasian	106 (68)	Separated	4 (3)
African-American	34 (22)	Divorced	11 (7)
Hispanic	7 (5)	Widowed	15 (10)
Other	8 (5)	Lives Alone	21 (14)
PAH Diagnosis		Currently employed	55 (35)
Idiopathic ("Primary")	63 (41)	Occupation	
Familial	2 (1)	Prof/Exec	22 (14)
Systemic Sclerosis	32 (21)	Manager	17 (11)
Other CVD	13 (8)	Clerical	45 (29)
Portal Hypertension	11 (7)	Skilled Labor	4 (3)
Anorectic Agent Use	7 (5)	Unskilled Labor	16 (10)
Other (i.e., HIV, PVOD)	27 (17)	Homemaker	12 (8)
		Other	9 (6)
WHO I, %	3 (2)	Not Available	30 (19)
WHO II, %	80 (52)	Substance Abuse	
WHO III, %	65 (42)	Prior Alcohol	11 (7)
WHO IV, %	7 (5)	Current Alcohol	0 (0)
		Prior Smoking	66 (43)
Mean RA pressure (mmHg)	8.7 ± 2.6	Current Smoking	12 (8)
Mean PA pressure (mmHg)	47 ± 17		
Cardiac output (liters/min)	4.9 ± 1.8		
PVR (dynes·sec·cm^-5^)	630 ± 425	**Treatments**	
**Symptoms**		Epoprostenol	50 (32)
Dyspnea	105 (68)	Bosentan	89 (57)
Fatigue, Weakness	55 (35)	Calcium Channel Blocker	52 (34)
Lightheadedness/Pre-syncope	31 (20)	Sildenafil	3 (2)
Chest Pain	31 (20)	Spironolactone	40 (26)
Cough	27 (17)	Other Diuretic(s)	76 (49)
Leg Pain	26 (15)	Warfarin	74 (48)
Jaw Pain	15 (10)	Digoxin	47 (30)
Depression	15 (10)	Oxygen, continuous	29 (19)
Anxiety	14 (9)	Oxygen, nocturnal	17 (11)
Palpitations	15 (10)	Oxygen, as needed	15 (10)
Diarrhea	22 (14)		
Abdominal Complaints	7 (6)		
Leg Weakness	7 (6)		
Difficulty Sleeping	3 (2)		

### Health-Related Quality of Life Scores

**Figure 1:**
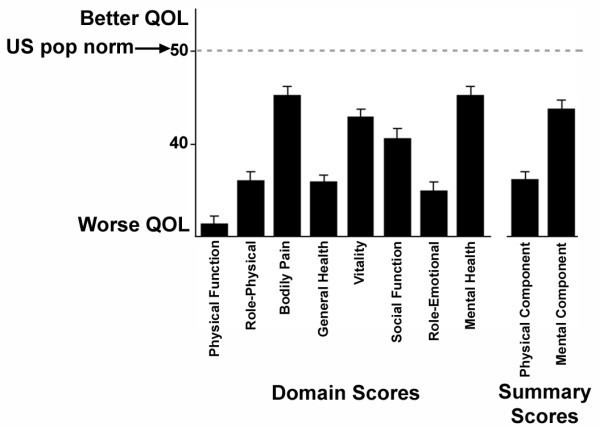
Health-Related Quality of Life Scores for Patients with Pulmonary Arterial Hypertension. Shown are mean (±SE) scores on each domain and summary component score of the SF36. Numerically higher scores indicate better health-related quality of life. All domain and summary scores are significantly lower than the US population normal score of 50 (p < 0.001 for each).

Patients with PAH had significantly depressed physical and mental health-related quality of life (Figure 1). On the SF-36, a "generic" measure of HRQOL applicable to a wide range of populations and disease states, HRQOL was impaired in every domain, representing a broad range of quality of life concepts (p < 0.001 for each). Mean scores were particularly depressed for the general health, physical functioning, role physical and emotional domains. In addition, the physical component summary (PCS) and mental component summary (MCS) scores, which broadly measure overall effects on HRQOL, were significantly decreased (p < 0.001 for each).

**Figure 2:**
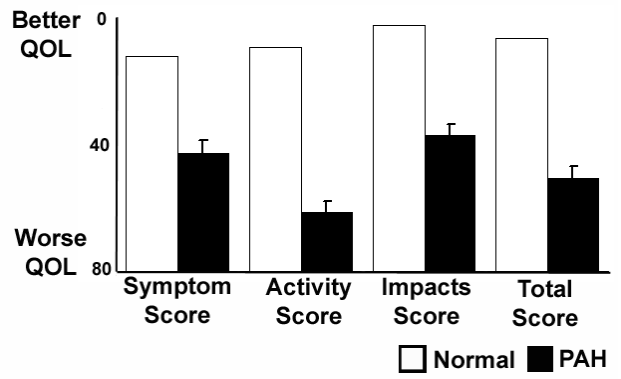
Respiratory-Disease Specific Health-Related Quality of Life Scores in Patients with Pulmonary Arterial Hypertension. Mean (±SE) scores on the Saint George's Respiratory Questionnaire for patients with pulmonary arterial hypertension are compared with the normal population. Numerically lower scores indicate better health-related quality of life. P < 0.0001 for the comparison between normal scores and PAH patients for each.

Quality of life was also assessed using a respiratory-disease specific instrument for comparison. Scores of patients with PAH were similarly abnormal on each component of the Saint George's Respiratory Questionnaire (Figure 2). Abnormally elevated scores (indicating a worse HRQOL) were seen in assessments of patient symptoms, activity, and the impact of disease on social and psychological function (p < 0.0001 for the comparison of each with normal). Further, PAH patients' mean total score, summarizing the effect of respiratory disease on overall health status, was markedly abnormal (p < 0.001).

### Factors Associated with Impaired Quality of Life

**Figure 3:**
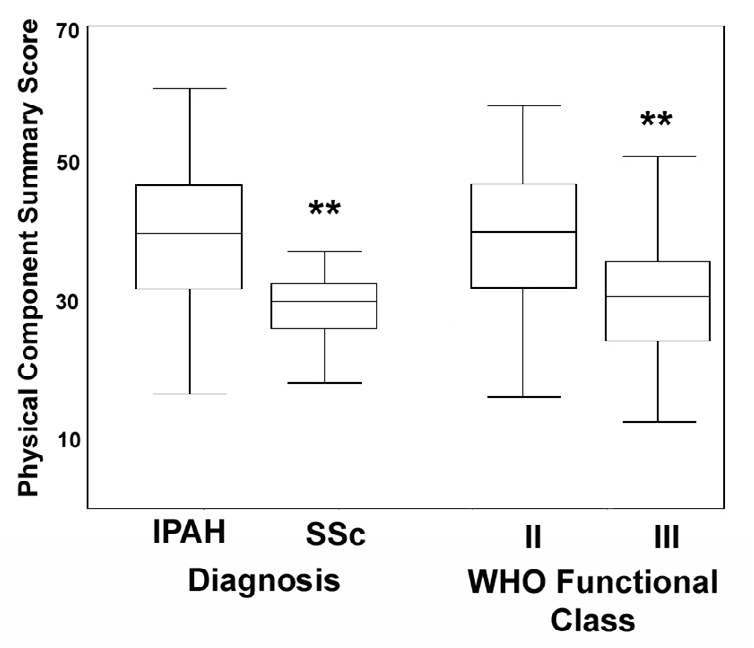
Box and whisker plots of scores on the physical and mental component summary measures of the SF36 according to PAH diagnosis and World Health Organization (WHO) Functional Class. ** indicates p < 0.0001 for the difference between patients with idiopathic pulmonary arterial hypertension (IPAH) and systemic sclerosis (SSc) related PAH, and for the difference between WHO Class II and III patients.

Idiopathic pulmonary arterial hypertension (IPAH) was associated with better health-related QOL than other causes of PAH (Figure 3). Physical component summary scores of the SF-36 were highest for patients with IPAH. Patients with disease associated with systemic sclerosis, on the other hand, had the poorest physical QOL scores. Mental component summary QOL scores differed only for patients with anorectic agent associated PAH, whose scores were lowest (p = 0.02; not shown). World Health Association (WHO) Class II patients had better physical component summary scores than those with Class III symptoms (p < 0.001); small numbers of patients with Class I and IV symptoms precluded further comparisons. Active employment was the only additional demographic variable associated with better physical component summary scores (p < 0.0001 for the comparison with patients who were not employed). HRQOL scores were not associated with gender, race or age. Correlation coefficients for age with physical and mental component scores were -0.148 (p = 0.08) and 0.05 (p = 0.58), respectively.

Certain symptoms were associated with worsened HRQOL. Chest pain and pre-syncope were each associated with poorer physical component scores (p ≤ 0.02 for each), generalized fatigue with lower physical and mental component scores (p ≤ 0.03, each). Common side effects of epoprostenol therapy (diarrhea, jaw pain) were not related to further impairment in HRQOL. Patients who reported abdominal discomfort had significantly worse physical and mental component summary scores, and the presence of abdominal tenderness on physical exam was associated with worsened MCS scores (p ≤ for each). The only additional finding on physical examination associated with poorer HRQOL was the presence of peripheral edema, with which lower physical component summary scores (p ≤ 0.0001) and mental component scores (p = 0.02) were seen.

**Figure 4:**
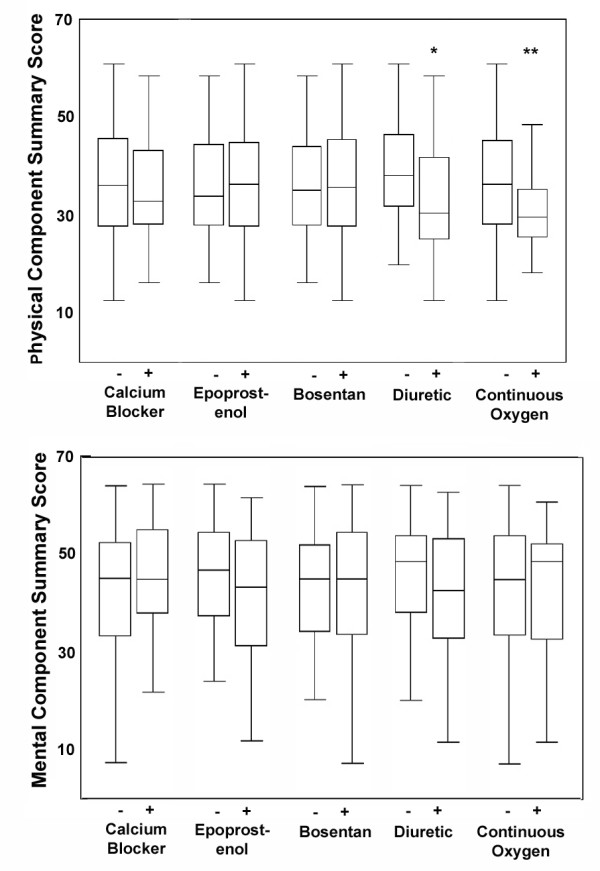
Box and whisker plots of scores on the physical and mental component summary measures of the SF36 according to treatments received at the time HRQOL assessment was performed. * indicates p ≤ 0.001 for the comparison between patients receiving or not receiving diuretics; ** p = 0.03 for the comparison between patients receiving or not receiving continuous oxygen therapy.

Therapies were also assessed. No differences in summary physical or mental component HRQOL scores were found between patients who were being treated with calcium channel antagonists, intravenous epoprostenol or bosentan at the time of HRQOL assessment (Figure 4). The use of diuretics or continuous oxygen was each associated with worsened physical summary scores. No differences were observed for the use/non-use of either warfarin or digoxin (not shown).

**Figure 5:**
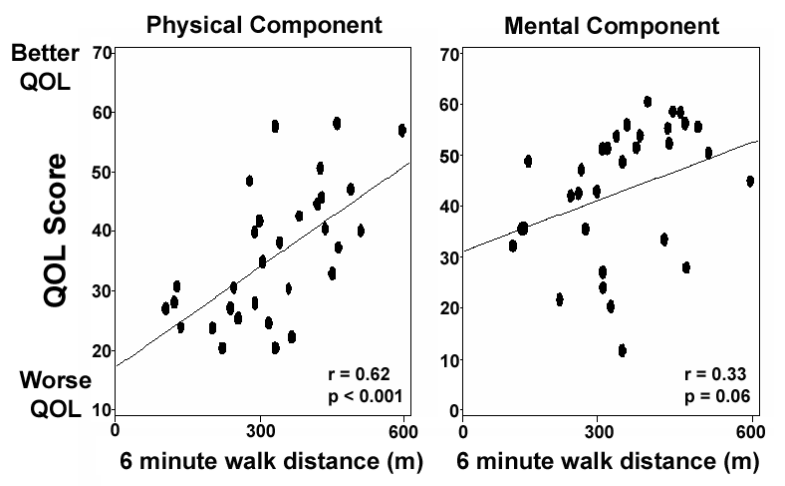
Correlation of six-minute walk distance and health-related quality of life scores. Y-axes indicate scores on the physical and mental component summary scores of the SF-36.

**Figure 6:**
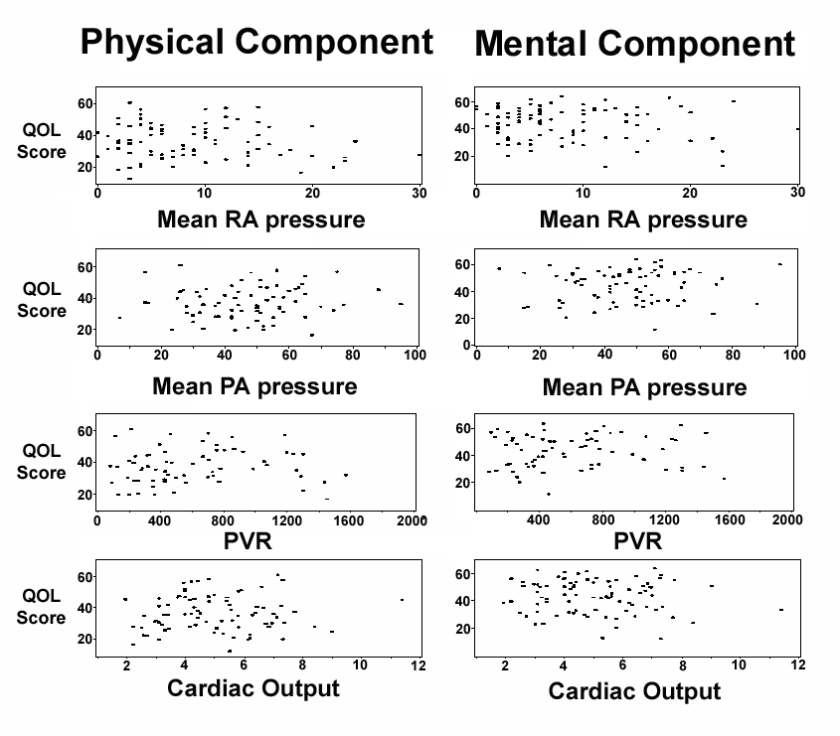
Lack of correlation of hemodynamic values and health-related quality of life scores. RA, right atrial pressure (mmHg); PA, mean pulmonary artery pressure (mmHg); PVR, pulmonary vascular resistance (dyne·sec·m^5^), Cardiac output = liters/minute. Y-axes indicate the physical and mental component summary scores of the SF-36.

The distance walked in six minutes was significantly correlated with the physical component summary HRQOL score and approached significance in correlation with the mental component summary scores (Figure 5). The degree of perceived exertion reported by patients during exercise testing (Borg dyspnea index; higher reported exertion indicating more severe dyspnea) was inversely correlated with the physical component score (correlation coefficient -0.46; p = 0.02). Oxyhemoglobin saturation at rest also correlated with physical component summary scores (correlation 0.24, p = 0.005), but not mental component scores. No correlation was observed between hemodynamic measurements and HRQOL scores (Figure 6). Neither physical nor mental component summary scores correlated with the mean right atrial or pulmonary artery pressures, cardiac output or pulmonary vascular resistance.

## Discussion

Changes in health-related quality of life have been reported in trials of medications for pulmonary arterial hypertension. Improvements over pre-treatment scores have been seen in association with increased exercise capacity resulting from various therapies [12-16]. Focused assessments of health-related quality of life itself, however, are lacking and a systematic evaluation of the factors influencing it has not been previously reported in this patient population. A recent cross-sectional study of 53 patients with PAH reported moderate to severe impairments in multiple domains of HRQOL, both physical and emotional [17].

In the present study we have assessed a large population of patients with PAH and have identified demographic, symptom and treatment factors associated with better or poorer health-related QOL. Significantly impaired HRQOL was found in each of the eight domains of the SF-36, representing those physical, social and emotional characteristics generally accepted as most directly affected by health and disease [18,19]. Similarly, impaired HRQOL was observed on all scales of a respiratory-specific assessment tool (the Saint George's Respiratory Questionnaire). We found HRQOL to be best for patients with idiopathic PAH and worst for those with systemic sclerosis, unrelated to the type of vasodilator therapy used, and to be correlated with functional but not hemodynamic assessments.

**Figure 7:**
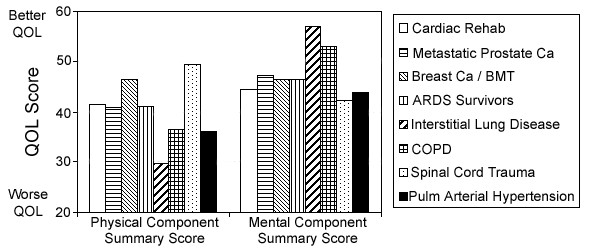
Comparison of health-related quality of life scores between disease states. Shown are mean population scores for the physical and mental component summary scores of the SF-36. Scores shown are derived from previous reports of cardiac rehabilitation [20], metastatic prostate cancer unresponsive to therapy [21], bone marrow transplantation for breast cancer [22], survivors of acute respiratory distress syndrome [23], interstitial lung disease [24], chronic obstructive lung disease [25] and spinal cord injury [26]. Previously published scores were normalized for comparison, as described in methods. Data shown for patients with pulmonary arterial hypertension are from the present study (as in Figure 1), shown here for comparison. Y-axis indicates the physical and mental component summary scores of the SF-36.

While we are not surprised to find reduced health-related quality of life in patients with PAH, the severity of impairment is remarkable. Impaired HRQOL – both physical and emotional – is associated with many chronic and physically debilitating conditions. The impairments observed in our population of PAH patients are as severe (and in many respects more so) than those reported in studies of patients with other severely debilitating and life-threatening conditions such as spinal cord injury, interstitial lung disease or cancer unresponsive to therapy (Figure 7) [20-26]. Indeed, Shafazand et al. found their patients with PAH unhappy enough with their condition as to be willing to accept a significant risk of death in exchange for a potential cure [17].

Several important clinical parameters were associated with the degree of impairment in quality of life we observed, including diagnosis and exercise capacity. In addition to their poorer overall prognosis [27,28], we found that patients with PAH associated with systemic sclerosis experience worse health-related QOL than patients with other forms of the disease. Despite clear improvements in hemodynamics and exercise capacity as compared to untreated patients, those with systemic sclerosis do not benefit to the same degree as patients with IPAH in response to PAH-specific therapies [29,30]. Further investigations are required to determine whether these patients derive less benefit in HRQOL in response to these therapies, or whether other specific interventions can result in improvements.

The results of a six-minute walk test have been shown to correlate with each the WHO functional Class and patient survival [31]. In our sample, the distance walked in six minutes correlated with health-related quality of life. Scores worsened as exercise capacity declined. Our data similarly indicated worse scores in patients with WHO Class III symptoms as compared with Class II. The low number of patients with WHO Class I or IV symptoms in our population, however, limits further comparisons. By contrast, it is interesting that our data did not indicate a correlation with hemodynamic values. This suggests that a patient's overall functional status is of greater importance than actual hemodynamic values in determining HRQOL. Such findings, if confirmed, emphasize the importance of such functional endpoints in clinical trials, and may help direct choices of therapy so as to bring about the greatest improvement in a patient's sense of well-being. These findings, however, require confirmation with larger data sets that allow for HRQOL assessments both before and after cardiac catheterization, the results of which frequently change in response to therapy.

That abdominal complaints were associated with impaired HRQOL may be an important finding among patients with PAH. While abdominal discomfort might be caused by impaired splanchnic perfusion due to poor cardiac performance, it can also (perhaps more commonly) result from any of a number of processes common in non-PAH patients (e.g. gastroesophageal reflux and peptic ulcer disease). Even if not the result of PAH's effect on circulatory function itself, the significant association of abdominal discomfort with HRQOL noted here warrants further investigation. Similarly, while swelling and edema are a common symptom and finding among patients with advanced PAH and cor pulmonale, the additional impairment in HRQOL associated with diuretic use emphasizes the need to be mindful of appropriate titration to the degree required to prevent significant fluid collection.

No difference was found in HRQOL between patients who were receiving calcium channel antagonists, bosentan or epoprostenol therapies at the time HRQOL assessments were made. We cannot determine from the present study whether this reflects differences in the factors that determined the choice of therapy, or if these treatments do indeed result in equivalent HRQOL. While it might be expected that the need for continuous intravenous therapy would be associated with worse HRQOL, the inconveniences of such treatment might be off-set by the resultant substantial and sustained improvements in exercise capacity. Further, HRQOL reflects an individual's satisfaction with his or her life as it is affected by health. As such, HRQOL will be affected differently on the basis of individual desires, perceptions and expectations [17]. Epoprostenol therapy requires a motivated patient willing and able to provide a high degree of self-care. Such therapy may thereby select for individuals whose HRQOL is influenced to a greater degree by a sense of control over one's disease and its treatments. Additional prospective studies will be required to further evaluate these possibilities, and to determine whether choice of therapy can be expected to influence a patient's HRQOL.

There are several limitations of this study. Retrospective collection of patient symptoms and diagnoses may have resulted in misclassification bias and thereby influenced the observed effects of various factors on HRQOL. Missing items on HRQOL questionnaires may not be equally distributed among patients, and could therefore serve as an additional source of bias. Multi-centered studies will be required to be sure that the associations with reported symptoms and treatments found here do not overly reflect the practice styles of the physicians at this single specialized center. Larger prospective studies are required to better assess potential differences in HRQOL resulting from different treatments.

There is no currently available instrument designed specifically for the assessment of health-related quality of life in patients with pulmonary arterial hypertension [5]. We have used the SF-36, a non-disease specific instrument and the most widely used worldwide in studies of many individual disease populations [9]. While we have used the SGRQ (an instrument aimed a patients with respiratory disease) as a means to compare and provide some validation of the results seen with the SF-36, this too is limited in its ability to evaluate many items contributing to HRQOL in patients with pulmonary arterial hypertension. The SGRQ was developed to assess patients with COPD. As such, many of its questions address symptoms not typical of pulmonary arterial hypertension (e.g. wheezing and productive cough). Further, other symptoms attributable to cor pulmonale and commonly faced by patients with pulmonary arterial hypertension are not addressed in the SGRQ. While several HRQOL instruments have been developed for patients with left heart failure, these too have significant limitations in application to other populations and have not been validated for use in patients with cor pulmonale. Indeed, pulmonary arterial hypertension represents a hybrid of respiratory and cardiac symptoms, signs and treatments. Pepke-Zaba and colleagues have recently reported on the development of a PAH-specific HRQOL questionnaire [32]. The availability of such an instrument specifically developed for evaluation of patients with pulmonary arterial hypertension will be an important contribution to future studies aimed at better understanding and improving HRQOL [5].

## Conclusion

Our study provides an initial systematic evaluation of health-related quality of life in a large population of patients with pulmonary arterial hypertension. We found profound impairment in all domains of HRQOL measured by both generic and respiratory-disease specific measures, and associations with assessments of functional status as well as specific demographic, symptom and treatment factors. As therapeutic options expand, the factors associated with impaired HRQOL identified here may suggest areas for further study and targeted intervention in efforts to improve outcomes for patients with pulmonary arterial hypertension.

## List of Abbreviations Used

PAH: Pulmonary Arterial Hypertension

HRQOL: Health-Related Quality Of Life

SF-36: Medical Outcomes Study 36 Short Form

SGRQ: St. George's Respiratory Questionnaire

PCS: Physical Component Summary

MCS: Mental Component Summary

IPAH: Idiopathic Pulmonary Arterial Hypertension

WHO: World Health Organization.

## Competing interests

Drs. Palevsky and Taichman have each served as consultants or on speaker's bureaus for Actelion Pharmaceuticals.

## Authors' contributions

DBT, JS, LH and HP designed and SK coordinated this study. JS, LH and CAC performed chart reviews. DBT, RG, JHF, JSS and JC interpreted the data and DBT wrote the manuscript.

## References

[B1] Rubin LJ (1997). Primary pulmonary hypertension. N Engl J Med.

[B2] Rich S, Dantzker DR, Ayres SM, Bergofsky EH, Brundage BH, Detre KM, Fishman AP, Goldring RM, Groves BM, Koerner SK (1987). Primary pulmonary hypertension. A national prospective study. Ann Intern Med.

[B3] D'Alonzo GE, Barst RJ, Ayres SM, Bergofsky EH, Brundage BH, Detre KM, Fishman AP, Goldring RM, Groves BM, Kernis JT (1991). Survival in patients with primary pulmonary hypertension. Results from a national prospective registry. Ann Intern Med.

[B4] Rubin LJ, Galie N (2004). Pulmonary arterial hypertension: a look to the future. J Am Coll Cardiol.

[B5] Hoeper MM, Oudiz RJ, Peacock A, Tapson VF, Haworth SG, Frost AE, Torbicki A (2004). End points and clinical trial designs in pulmonary arterial hypertension: clinical and regulatory perspectives. J Am Coll Cardiol.

[B6] Highland KB, Strange C, Mazur J, Simpson KN (2003). Treatment of pulmonary arterial hypertension: a preliminary decision analysis. Chest.

[B7] McGoon M, Gutterman D, Steen V, Barst R, McCrory DC, Fortin TA, Loyd JE (2004). Screening, early detection, and diagnosis of pulmonary arterial hypertension: ACCP evidence-based clinical practice guidelines. Chest.

[B8] Barst RJ, McGoon M, Torbicki A, Sitbon O, Krowka MJ, Olschewski H, Gaine S (2004). Diagnosis and differential assessment of pulmonary arterial hypertension. J Am Coll Cardiol.

[B9] Ware JEKMDJE (2000). How to Score Version 2 of the SF-36 Health Survey..

[B10] Jones PW, Quirk FH, Baveystock CM, Littlejohns P (1992). A self-complete measure of health status for chronic airflow limitation. The St. George's Respiratory Questionnaire. Am Rev Respir Dis.

[B11] Jones PW, Spencer S, Adie S (2003). The St. George's Respiratory Questionnaire Manual, Version 2.1.

[B12] Olschewski H, Simonneau G, Galie N, Higenbottam T, Naeije R, Rubin LJ, Nikkho S, Speich R, Hoeper MM, Behr J, Winkler J, Sitbon O, Popov W, Ghofrani HA, Manes A, Kiely DG, Ewert R, Meyer A, Corris PA, Delcroix M, Gomez-Sanchez M, Siedentop H, Seeger W (2002). Inhaled iloprost for severe pulmonary hypertension. N Engl J Med.

[B13] Barst RJ, Rubin LJ, Long WA, McGoon MD, Rich S, Badesch DB, Groves BM, Tapson VF, Bourge RC, Brundage BH (1996). A comparison of continuous intravenous epoprostenol (prostacyclin) with conventional therapy for primary pulmonary hypertension. The Primary Pulmonary Hypertension Study Group. N Engl J Med.

[B14] Archibald CJ, Auger WR, Fedullo PF, Channick RN, Kerr KM, Jamieson SW, Kapelanski DP, Watt CN, Moser KM (1999). Long-term outcome after pulmonary thromboendarterectomy. Am J Respir Crit Care Med.

[B15] Sastry BK, Narasimhan C, Reddy NK, Raju BS (2004). Clinical efficacy of sildenafil in primary pulmonary hypertension: a randomized, placebo-controlled, double-blind, crossover study. J Am Coll Cardiol.

[B16] Simonneau G, Barst RJ, Galie N, Naeije R, Rich S, Bourge RC, Keogh A, Oudiz R, Frost A, Blackburn SD, Crow JW, Rubin LJ (2002). Continuous subcutaneous infusion of treprostinil, a prostacyclin analogue, in patients with pulmonary arterial hypertension: a double-blind, randomized, placebo-controlled trial. Am J Respir Crit Care Med.

[B17] Shafazand S, Goldstein MK, Doyle RL, Hlatky MA, Gould MK (2004). Health-related quality of life in patients with pulmonary arterial hypertension. Chest.

[B18] Ware JEJ, Gandek B (1998). Overview of the SF-36 Health Survey and the International Quality of Life Assessment (IQOLA) Project. J Clin Epidemiol.

[B19] Ware JEJ, Sherbourne CD (1992). The MOS 36-item short-form health survey (SF-36). I. Conceptual framework and item selection. Med Care.

[B20] Cohen RA, Moser DJ, Clark MM, Aloia MS, Cargill BR, Stefanik S, Albrecht A, Tilkemeier P, Forman DE (1999). Neurocognitive functioning and improvement in quality of life following participation in cardiac rehabilitation. Am J Cardiol.

[B21] Albertsen PC, Aaronson NK, Muller MJ, Keller SD, Ware JEJ (1997). Health-related quality of life among patients with metastatic prostate cancer. Urology.

[B22] Hann DM, Jacobsen PB, Martin SC, Kronish LE, Azzarello LM, Fields KK (1997). Quality of life following bone marrow transplantation for breast cancer: a comparative study. Bone Marrow Transplant.

[B23] Davidson TA, Caldwell ES, Curtis JR, Hudson LD, Steinberg KP (1999). Reduced quality of life in survivors of acute respiratory distress syndrome compared with critically ill control patients. JAMA.

[B24] Chang JA, Curtis JR, Patrick DL, Raghu G (1999). Assessment of health-related quality of life in patients with interstitial lung disease. Chest.

[B25] Mahler DA, Mackowiak JI (1995). Evaluation of the short-form 36-item questionnaire to measure health-related quality of life in patients with COPD. Chest.

[B26] Lucke KT, Coccia H, Goode JS, Lucke JF (2004). Quality of life in spinal cord injured individuals and their caregivers during the initial 6 months following rehabilitation. Qual Life Res.

[B27] Kawut SM, Taichman DB, Archer-Chicko CL, Palevsky HI, Kimmel SE (2003). Hemodynamics and survival in patients with pulmonary arterial hypertension related to systemic sclerosis. Chest.

[B28] Kuhn KP, Byrne DW, Arbogast PG, Doyle TP, Loyd JE, Robbins IM (2003). Outcome in 91 consecutive patients with pulmonary arterial hypertension receiving epoprostenol. Am J Respir Crit Care Med.

[B29] Badesch DB, Tapson VF, McGoon MD, Brundage BH, Rubin LJ, Wigley FM, Rich S, Barst RJ, Barrett PS, Kral KM, Jobsis MM, Loyd JE, Murali S, Frost A, Girgis R, Bourge RC, Ralph DD, Elliott CG, Hill NS, Langleben D, Schilz RJ, McLaughlin VV, Robbins IM, Groves BM, Shapiro S, Medsger TAJ (2000). Continuous intravenous epoprostenol for pulmonary hypertension due to the scleroderma spectrum of disease. A randomized, controlled trial. Ann Intern Med.

[B30] Rubin LJ, Badesch DB, Barst RJ, Galie N, Black CM, Keogh A, Pulido T, Frost A, Roux S, Leconte I, Landzberg M, Simonneau G (2002). Bosentan therapy for pulmonary arterial hypertension. N Engl J Med.

[B31] Miyamoto S, Nagaya N, Satoh T, Kyotani S, Sakamaki F, Fujita M, Nakanishi N, Miyatake K (2000). Clinical correlates and prognostic significance of six-minute walk test in patients with primary pulmonary hypertension. Comparison with cardiopulmonary exercise testing. Am J Respir Crit Care Med.

[B32] Doughty NMKSPMDCFPZJ (2005). The CAMPHOR:  Correlations with Objective Measures of Severity of Pulmonary Hypertension.. Proceedings of the American Thoracic Society.

